# Transcranial electrical evoked muscle potentials for pediatric neurosurgery: scoping review of stimulation techniques and success rates

**DOI:** 10.1007/s00381-024-06739-4

**Published:** 2025-01-08

**Authors:** Axel Fudickar, Kai Berndt, Klaus Novak

**Affiliations:** 1 Department of Anesthesiology and Intensive Care Medicine, Christian-Albrechts-University, University Hospital Schleswig-Holstein, Campus Kiel, Arnold-Heller-Str. 3/R3, 24105 Kiel, Germany; 2 Department of Neurosurgery, Medical University of Vienna, Währinger Gürtel 18-20, Wien, A-1090 Austria

**Keywords:** Electromyogram, Transcranial motor evoked potentials, Pediatric neurosurgery, Stimulation technique

## Abstract

**Purpose:**

The background of this scoping review is that pediatric neurosurgery in the vicinity of motor pathways is associated with the risk of motor tract damage. By measuring transcranial electrical evoked potentials in muscles (electromyogram) or from the spinal cord (epidural D-wave) functional disorders and impending damage can be detected during surgery and countermeasures can be initiated. The objective was to summarize stimulation techniques of transcranial electrical stimulation and the success rate of motor evoked potentials exclusively in children undergoing neurosurgery.

**Methods:**

The data source was a literature search for reports meeting the suitability criteria (original articles and case series including motor evoked potentials and pediatric neurosurgery).

**Results:**

Twenty-four articles meeting suitability criteria were retrieved. The most common primary electrode positions for electrical stimulation were at C3 vs. C4 and C1 vs. C2 according to the 10–20-system of EEG. Single trains of 1 to 9 pulses with voltages from 160 to 900 V and pulse durations from 50 to 500 µs were applied for voltage-controlled stimulation. Interstimulus intervals ranged from 0.1 to 9.9 ms. Signals were filtered with high-pass filters between 1.5 and 300 Hz and low-pass filters between 500 and 5000 Hz. The overall rate of successful stimulation and measurement was 90.5% (*N* = 769).

**Conclusion:**

A broad range of stimulation parameters was used for transcranial electrical evoked potentials. Measurable potentials were obtained in most patients. Consideration of safety precautions is an important implication to avoid adverse events by application of high voltage to the motor cortex.

## Introduction

**Fig. 1:**
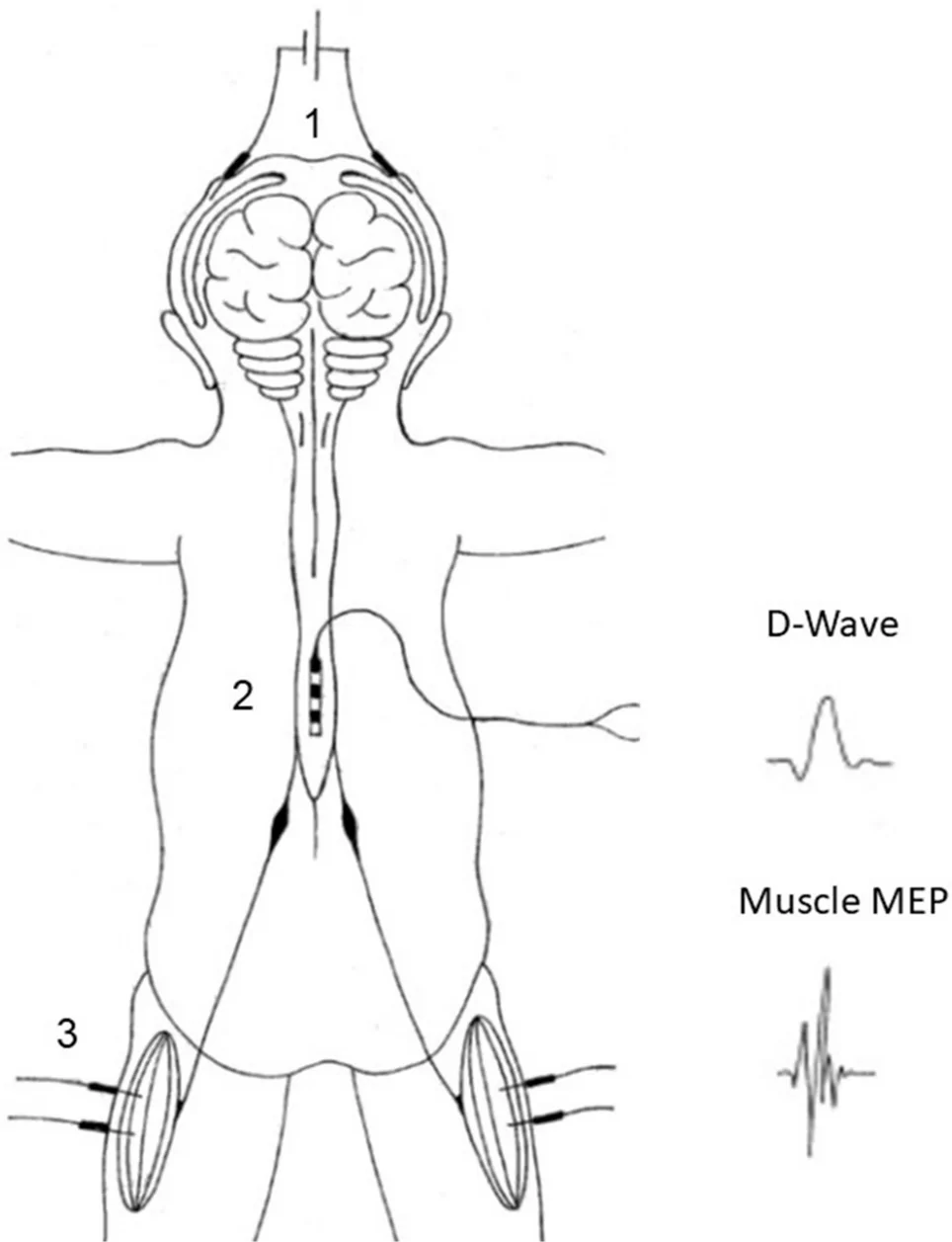
Schematic depiction of motor evoked potentials and D-wave after transcranial electrical stimulation of the motor cortex. Legend: 1 = Transcranial electrical stimulation using straight transdermal subcutaneous needle electrodes., 2 = D-wave recording by an epidural electrode., 3 = Motor evoked potential (MEP) recording using straight transdermal intramuscular needle electrodes

Pediatric neurosurgery in the vicinity of motor pathways is associated with the risk of motor tract damage. In the course of pediatric brain and spinal cord surgery, continuous measurement of motor pathway function can be provided by intraoperative neuromonitoring (IONM) using transcranial electrical evoked potentials (tceMEP). IONM is defined as monitoring of the nervous system during surgery. IONM is indicated if a pathological finding of IONM is expected to prompt an adjustment of the surgical procedure aiming at improving of the neurological outcome. tceMEP are electrical potentials obtained from muscles, e.g., by inserting needle electrodes (electromyogram, EMG) or directly from the spinal cord by inserting epidural electrodes (D-wave) after transcranial electrical stimulation of the motor cortex (Fig. 1) [1, 2]. Monitoring tceMEP in limb muscles provides functional monitoring of motor pathways including the corticospinal tract, spinal roots, and peripheral nerves, while D-wave recording is limited to monitoring the corticospinal tract [3, 4]. Additional measurement of somatosensory potentials after stimulation of the tibial nerve (TSEP) or median nerve (MSEP) can extend monitoring to spinal sensory tracts, but monitoring of TSEP or MSEP alone is insufficient to avoid postoperative motor function deficits with acceptable reliability [4–6]. It has been reported that tceMEP predict postoperative motor deficits with sufficient sensitivity.

If impairment of motor nerve or spinal tract function occurs, feedback from the neurophysiologist to the neurosurgeon can prompt a change of the surgical procedure aiming at reduction of the risk of irreversible iatrogenic injury to motor pathways [7]. The effectiveness of tceMEP to improve neurological outcome has been shown in numerous studies. Monitoring tceMEP reduces for example the incidence of accidental injury to the spinal cord during surgery for intramedullary spinal cord tumors and is consequently recommended for spinal tumor surgery [7, 8].

Based on early experiences and physiological considerations of immaturity of neuronal myelinization and lack of synchrony of action potential propagation, it has been argued that tceMEP could not be evoked reliably in young children at an age of less than 21 months [9–14].

This seems to be confirmed by the fact that baseline stimulus thresholds in infants are higher than in adults [15]. However, tceMEP have been repeatedly recorded from patients younger than 12 months, thus encouraging routine use of tceMEP in small children [16, 17]. Numerous case reports and studies reporting about tceMEP in pediatric neurosurgery are now available including different stimulation techniques and reporting different success rates. However, a systematic overview over stimulation techniques and success rates is lacking, but would be particularly useful for novices and clinicians without special scientific background in clinical neurophysiology. Those technical considerations are predominantly important in neurosurgical patients, because neurological impairment due to the pathological findings leading to neurosurgery may hamper successful measurement of tceMEP. In this review, stimulating parameters of transcranial electrical stimulation for motor evoked potentials in pediatric neurosurgery were reviewed together with success rates and safety considerations. Not only specialized neurophysiologists and technicians but also pediatric neurosurgeons, neuropediatricians, child psychologists, and pediatric neuroanesthesiologists perform intraoperative monitoring including tceMEP. Thus, this scoping review addresses all occupational groups involved or interested in the application of this technique. Pediatric neurosurgeons not directly involved in the practical implementation of intraoperative neuromonitoring are also target readers, because close cooperation between pediatric neurosurgeons and the neurophysiologists is mandatory during tceMEP and is facilitated by detailed technical knowledge of all professional groups involved.

## Methods

**Fig. 2:**
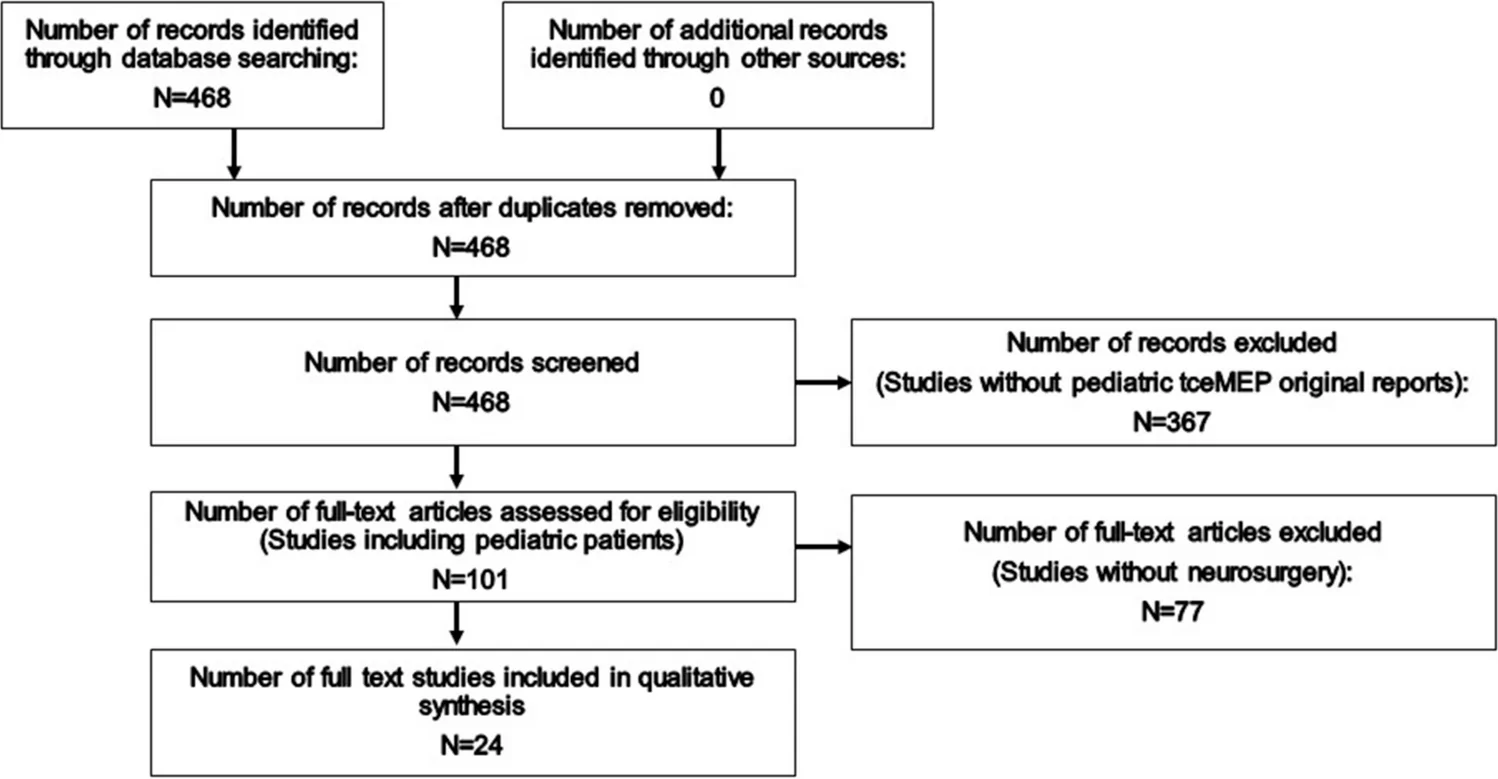
Flow chart of the search and selection strategy. Legend: Schematic flow chart of the literature search and selection strategy: Vertical arrows represent the process leading to the articles selected for evaluation, horizontal arrows represent the exclusion of articles not relevant for evaluation

Criteria for study suitability were original articles or case series of intraoperative motor evoked potentials in children undergoing neurosurgical procedures. A PubMed and Cochrane database literature search using the terms “intraoperative motor evoked potentials children” yielded 468 articles with potential relevance (last accessed in November 2024). From the abstracts of these articles, 367 abstracts of studies without pediatric patients were excluded. The remaining 101 full-text articles were assessed for suitability by screening for original articles and case series including neurosurgery with tceMEP (Fig. 2). AF screened the records and retrieved reports for suitability. KB and KN independently reviewed the records and reports. No automation tools were used in the process.

The retrieved articles were screened for ages of participants, number of participants, number of patients with successfully elicited tceMEP, stimulating electrode montage, current and voltage amplitudes, train parameters, filter settings, and the applied monitoring systems using electronic forms. Where applicable, further information about criteria for successful measurement, modification of stimulation parameters in case of failure to obtain tceMEP, safety precautions, alarm criteria, and age dependency of successful tceMEP were obtained. Publication dates and numbers of investigated subjects were retrieved for each included article.

For data analysis, the commercially available software Excel (Microsoft Office 2007, Microsoft Corporation, USA) was utilized.

## Results

The literature search yielded 24 original articles and case series reporting on motor evoked potentials in pediatric neurosurgery (Fig. 2).

### Equipment and recording parameter

A huge variety of monitoring devices was reported in the analyzed articles. In 24 publications, 20 different systems were used. The most frequently used devices were Eclipse (Axon Systems, Hauppauge, NY or Medtronic, Minneapolis, MN, 6 publications) and Digitimer (Digitimer Co., Welwyn Garden City, UK, 4 publications). One article did not contain information about the monitoring equipment.

Most authors derived tceMEP from limb muscles using needle electrodes. Two authors obtained D-wave spinal potentials after transcranial electrical stimulation [9, 18]. Signals were filtered with high-pass filter frequencies ranging from 1.5 to 300 Hz and low-pass filter frequencies ranging from 853 to 5000 Hz. The mean high-pass filter frequency was 107.7 Hz (112.3 Hz), and the mean low-pass filter frequency was 2205.9 Hz (1351.3 Hz) (mean (standard deviation), Table 1) [4, 5, 9, 10, 16–18, 20–22, 24, 26, 28–30, 32, 33].

**Table 1:** Filter settings and equipment for pediatric transcranial electrical motor evoked potentials if provided by the authors

First author	Year	LFF (Hz)	HFF (Hz)	Equipment
Szelenyi [9]	2003	1,5	853	Digitimer D-180, Digitimer Co., Welwyn Garden City, UK
Fulkerson [15]	2011			Digitimer MultiPulse Stimulator model D185—Mark IIa (Digitimer Ltd.Endeavor CR recording device (CareFusion)
Patel [19]	2012			Digitimer MultiPulse Stimulator model D185—Mark IIa (Digitimer Ltd. Hertfordshire, UK)
Jackson [4]	2014			Axon Epoch XP, Axon Systems; Nicolet Endeavor, Viasys Healthcare, Digitimer D185, Digitimer Corp
Cheng [5]	2014			Digitimer D185 constant-voltage stimulator (Digitimer Ltd.)
Bozinov [20]	2015	100	3000	ISIS system, INOMED, Germany
McIntyre [21]	2016	30		Medtronic NIMNim Eclipse 3.5 (Medtronic Inc., Minneapolis, MN)
Barzilai [22]	2016			Axon XP/Axon Eclipse, Axon Systems, Hauppauge, NY, or NIM Eclipse Medtronic, Minneapolis, MN
Senköylü [23]	2017			Nicolet Endeavor CR™ (Viasys Healthcare, Nicolet Biomedical, Madison, USA)
Motomura [24]	2018			Neuromaster MEE1200, Nihon Kohden, Tokyo, Japan
Dias [25]	2018			ISIS IONM (Inomed Medizintechnik GmbH)
Yi [17]	2019			NIMEclipse NS system (Medtronic Xomed Inc.)
Aydinlar [26]	2019	5	2000	32-channel Cadwell Cascade Elite (Cadwell Industries, Kennewick, WA)
Roth [27]	2020			NIM Eclipse (Metronic, Minneapolis, MN)
Nair [28]	2021	10	500	Cadwell, Endeavour (Nicolet Biomedical Inc), USA
Takatani [29]	2021	300	3000	Neuromaster MEE-1232, Nihon Kohden, Japan
Udayakumaran [30]	2021			Xeltek Protektor 32 IOM System, Natus Neurology Medical Inc., Middleton, WI, USA
Herta [16]	2022	200	1500	NIM-Eclipse® monitoring system ver. 3.5.354 (Medtronic XOMED Inc., Memphis, TN, USA)
Antkowiak [18, 31]	2022			ISIS Xpert Plus System (Inomed Polska, Rokitnica, Poland)
San-Juan [32]	2023	100	5000	NIM Eclipse E3, Medtronic Co, Minneapolis
Sasaki [33]	2023	300	3000	Neuromaster MEE-2032G1; Nihon Kohden
McGrath [34]	2024	30	1000	Cadwell Cascade (Cadwell Laboratories)

*LFF* low frequency filter, *HFF* high frequency filter

### Stimulation

Most primary stimulation electrode positions were at C3 vs. C4 (*N* = 12) and C1 vs. C2 (*N* = 5). One author used a position 2 cm anterior of C3 vs. C4 and one author used a position 1 cm anterior of C1 vs. C2 [21, 27]. Positions were published according to the international 10–20-system of EEG measurement for electrode positions [35]. Stimulation electrodes were corkscrew electrodes or needle electrodes. Needle electrodes were inserted tangentially in relationship to the skin surface predominantly in small infants with open fontanels to avoid damage of deeper structures.

Most authors used constant voltage pulses. Voltages of pulses ranged from 100 to 900 V. The mean minimal voltage was 277.2 V (119.8) and the mean maximal voltage was 531.1 V (212.1 V) (mean (standard deviation)). Stimulation techniques were slow-charge stimulation (200 to 500 µs pulse width) or fast-charge stimulation (50 to 75 µs pulse width). Stimulation was in most publications performed using one pulse train including four to nine single alternating pulses with a median minimal train count of 5 (2–9, 5, (range, median)) and a median maximal train count of 7 (5–9, 7, (range, median)). Interstimulus interval ranged from 0.1 to 9.9 ms. The mean minimal interstimulus interval was 2.2 ms (0.7 ms) and the mean maximal interstimulus interval was 3.65 ms (2.8 ms) (mean (standard deviation), Table 2). When constant voltage pulses are applied, automatic limitation of the resulting current is mandatory.

**Table 2:** Montage, voltages, impulse widths, and train parameter applied for pediatric transcranial electrical motor evoked potentials with voltage-controlled stimulation if provided by the authors

First author	Year	tceMEP position	Vmin (V)	Vmax (V)	ID (µs)	Train min	Train max	ISI (ms) min	ISI (ms) max
Szelenyi [9]	2003	C3/C4, C1/C2			50	5.00	5.00	4.00	4.00
Fulkerson [15]	2011	C3/C4	321	746	50	4.00	4.00	3.00	3.00
Patel [19]	2012		387	600	50	4	7	3	3
Jackson [4]	2014	C1/C2	250	500	50	2.00	5.00	1.00	3.00
Cheng [5]	2014	Motor Cortex	275	625				0.10	9.90
McIntyre [21]	2016	C1/C2**			300	9	9	2.00	4.00
Senköylü [23]	2017	M3-Mz6/ M6-Mz6	176	400	1000	5	10	4	4
Yi [17]	2019	C3/C4	290	900	75	8	8	2.00	2.00
Aydinlar [26]	2019	C3/C4	310	740	500	6	7	4.00	4.00
Roth [27]	2020	C3/C4*				5	7	3	3
Gisi [36]	2020		200	250		4	4	2.00	2.00
Nair [28]	2021	C1/C2	180	320	75	5.00	7.00		
Takatani [29]	2021	C3/C4	500	500				2.00	2.00
Udaya-kumuran [30]	2021	Fc3/Fc4	200	500	50	8	8		
San-Juan [32]	2023	C3/C4			500	5	7	2	2
Sasaki [33]	2023	C3/C4	500	500		5	5	2	2
McGrath [34]	2024	C3/C4	100	500	50	4	8	1	2

*tceMEP* transcranial electrical evoked potentials, *ID* impulse duration, *ISI* interstimulus interval, *min* minimal, *max* maximal, *2 cm in front of C3/C4, **1 cm in front of C3/C4

Seven authors reported about constant current stimulation. Applied current strength was a maximal 220 mA with an impulse duration of 500 µs. The mean minimal current strength was 55.9 mA (44.9 mA), and the mean maximal current strength was 202.1 mA (32.7 mA) (mean (standard deviation), Table 3) Train counts ranged from 1 to 9 with an interstimulus interval ranging from 2 to 4 ms (Table 3) [4, 5, 9, 16–18, 20–22, 24, 26, 28–33].

**Table 3:** Montage, currents, impulse widths, and train parameter applied for pediatric transcranial electrical motor evoked potentials with current-controlled stimulation if provided by the authors

First author	Year	tceMEP Position	Imin (mA)	Imax (mA)	ID (µs)	Train min	Train max	ISI (ms) min	ISI (ms) max
Szelényi [9]	2003	C3/C4, C1/C2		200	500	1	1	4	4
Bozinov [20]	2015	C3/C4	15	220	500	3	7	2	2
Barzilai [22]	2016		100	200	500	5	5	3	3
Motomura [24]	2018		80	180	500	5	5	2	2
Dias [25]	2018		40	220					
Herta [16]	2022	C1/C2,C3/C4	101 (20)	200	500	5	9	4	4
Antkowiak [18, 31]	2022	C1/C2,C3/C4	90	140	500	4	6		

tceMEP transcranial electrical evoked potentials, ID impulse duration, ISI interstimulus interval, min minimal, max maximal.

### tceMEP measurement success rate

The mean tceMEP measurement success rate was good (85.9% (21.2), mean (standard deviation)) and increased from earlier studies to more recent reports (Table 4) [4, 5, 9, 16, 17, 20, 21, 24, 26, 28–31, 33]. The success rate was 203 of 223 patients (91.0%) for primary stimulation at C1/C2 and 310 of 327 patients (94.8%) for primary stimulation at C3/C4. No complication was reported in any of the studies included in this review.

**Table 4:** Patient ages, patient numbers, and success rates for transcranial electrical motor evoked potentials if provided by the authors

First author	Year	Age	Age max	*N*	n/N Success	% success
Szelenyi [9]*	2003	8 m–36 m	36 m	19	7	36.8
Fulkerson [15]**	2011	5 m–31 m (16.8 M)	31 m	10	10	100
Patel [19]**	2012	4 y–16 y	16 y	6	4	66
Jackson [4]**	2014	6 m–5 y	5 y	6	6	100
Cheng [5]**	2014	2 y–14 y	14 y	12	12	100
Bozinow [20]**	2015	5 m–15 y [5.5 y]	15 y	21		100
McIntyre [21]**	2016	3 w–71 m	71 m	117	110	94
Barzilai [22]**	2016	1 y–17 y	17 y	21		
Roth [27]**	2020	3 m–207 m	207 m	42		
Senköylü [23]	2017	< 11 y	11 y	43	43	100
Motomura [24]**	2018	0 y–15 y [8 y]	15 y	60	41	68
Dias [25]**	2018	2 y–14 y (8.4 y)	14 y	17	17	100
Yi [17]**	2019	39 d–87 d (72.8 d)	87 d	25	24	96
Aydinlar [26]**	2019	3 m–12 m (5.82 m ± 3.45 m)	12 m	15	15	100
Gisi [36]**	2020	2 d–3 m	3 m	2	1.00	50
Nair [28]**	2021	3 m–24 m (17 m ± 5.8 m)	24 m	100	87	87
Takatani [29]**	2021	6.0 ± 5.1 y		31		87
Udayakumaran [30]**	2021	1 m–9 m (7.5 m)	9 m	205		99.4
Herta [16]	2022	13 w–49 w (33 w)	49 w	22	21	95.5
Antkowiak 1 [31]*	2022	1.3 y–18.8 y (8.6 y)	18.8 y	23	23	100
Antkowiak 2 [18]**	2022	1 y–17.7 y	17.7 y	16		
San Juan [32]**	2023	1 y–17 y	17 y	32		
Sasaki [33]**	2023	< 15 y (8.9 y ± 4.9)	15 y	15		37.5
McGrath [34]**	2024	2 y–17 y [10.4]	17 y	20	20	100

y years, m months, w weeks, d days, max maximal, (mean), [median], *D-wave, **Motor evoked potentials

### Criteria for successful measurement of tceMEP

In most cases, muscle potential amplitudes and number of muscles with measurable tceMEP were used as success criteria. Five authors regarded an amplitude of muscle potentials > 50 µV as a sufficient criterion for successful stimulation and measurement [16, 21, 26, 28, 37]. Two authors limited this criterion to patients with tceMEP > 50 µV in at least 50% of measured muscles [16, 21], and one author limited the criterion to tcMEP > 50 µV in at least one muscle group [28].

### Modification of primary stimulation to improve tcMEP

Two authors published experiences and recommendations for modifications of primary stimulation to improve tcMEP due to failure to obtain sufficient tcMEP with standard settings. Increasing the pulse voltage is one means to overcome this problem. However, adapting the time course of transcranial electric stimuli by increasing the number of stimuli and pulse durations or implementation of double train technique may render tceMEP feasible without applying a higher stimulus intensity. This optimization method has been described as temporal facilitation [16]. Obtaining tceMEPs from distal muscles with more innervation than proximal muscles may also improve the success rate [16]. If inhalational anesthesia is used, a change to total intravenous anesthesia (TIVA) can improve tceMEP measurement [17]. Hence, most authors used total intravenous anesthesia based on propofol and an opioid to facilitate tceMEP measurement.

### Age dependency of successful measurement

In the study published by Szelenyi et al. in 2003, a transcranial electrical evoked D-wave was measurable in only 7 of 19 children at an age between 8 and 36 months during intraoperative neuromonitoring of intramedullar spinal tumor surgery. The youngest child with measurable D-waves was 21 months old, thus suggesting a limited success rate of tceMEP in very young children. Potential obstacles discussed in this report were prolonged corticospinal tract maturation with high individual variability. However, effects of intramedullar tumors on corticospinal tract function were not excluded [9].

In a later publication, successful measurement of tceMEP was reported for children between 6 months and 5 years with myelomeningocele repair [4]. However, the two different methodologies tceMEP and D-wave cannot be directly compared, even if both methods include transcranial electrical stimulation, and the observed age dependence was confirmed in a further study [26]. However, in later studies, monitoring of tceMEP was extended to even younger children. tceMEP were successfully measured in 24 of 25 infants less than 3 months of age. Only one infant with preoperative lethargy and weakness in all extremities could not be monitored by tceMEP due to failure to elicit muscle potentials [17]. Moreover, no age dependency was found in a large retrospective analysis of 100 children [28]. tceMEP were also present in upper extremities in all of 22 infants and in lower extremities in 21 out of 22 infants in a report on tceMEP in infants with an age of less than 1 year. The only infant without measurable muscle potentials suffered from preoperative paresis. Patient age revealed only a weak significant correlation to stimulation intensity (*r* = − 0.381, 95% CI − 0.628 to − 0.065, *p* = 0.020*) in a univariate analysis that could not be reproduced in multivariate analysis (95% CI − 2.441 to 0.066, *p* = 0.063) of the same data set [16].

## Discussion

The main results of our review are:

1. A huge variety of suitable devices and filter settings for tceMEP was found in the analyzed articles.
2. Most authors used voltage control stimulation and provided stimulation electrode positions, voltages, impulse widths, and train parameter.
3. Success rates were high even in investigations including very young children.

### Equipment and recording

The huge number of different monitoring devices used by the authors reflects the broad availability of reliable monitoring devices for the measurement of tceMEP. Most authors published data for stimulation electrode positions (montage), stimulation intensity, time course of stimulation, and filter settings for gathering muscle and spine potentials (Tables 2 and 3), thus providing a broad spectrum of feasible stimulation and measurement techniques.

D-wave spinal potentials after transcranial electrical stimulation have been estimated as more reliable than muscle potentials due to the advantage of measuring potentials directly from the spinal cord [9]. However, most authors reported successful measurements with muscle needle electrodes, thus taking advantage of the extended pathway monitored by muscle MEPs (corticospinal tract for D-wave versus corticospinal tract and other descending systems for muscle MEPs) and the higher short-term predictive value of muscle MEP in comparison to D-wave. Unlikely from muscle tceMEPs, which can be used for both cranial and spinal surgery, D-wave monitoring applies exclusively to spinal surgery and mostly to spinal cord tumor surgery.

When reported, most authors choose an amplitude of muscle potentials > 50 µV as a sufficient criterion for successful stimulation and measurement and the rates of thus defined successful measurement were very good in most studies [16, 21, 26, 28, 38].

### Stimulation

Electrode positions C3 vs. C4 and C1 vs. C2 can be used to elicit tceMEP in infants and children according to the guidelines of the ACNS [39]. The guidelines also describe stimulation over electrodes at Cz vs. Fz for more reliable stimulation of the motor pathways to the lower limbs bilaterally with a single stimulus, but this montage has not been described in the reviewed papers. Montage of stimulus electrodes has important implications for the depth of stimulation, and depth of stimulation may, particularly for brain surgery, have an impact on the risk of falsely negative tceMEP and potential side effects. Due to their proximity to the jaw muscles and the trigeminal nerve, stimulation at C3 vs. C4 electrodes can elicit stronger biting movements. Anatomically, C3 vs. C4 comprises more motor cortex areas, but stimulation may be hampered by more electric field inhomogeneity and loss of electrical energy in comparison to C1 vs. C2 due to the closer proximity of C1 vs. C2 in comparison to C3 vs. C4. Hence, C1 vs. C2 electrodes are preferable in principle [40, 41]. However, C3 vs. C4 montage was preferred by most authors in our review possibly due to anticipation of a more reliable stimulation effect in both upper and lower limbs with one stimulus train than C1 vs. C2-montage. The overall success rate was slightly higher for primary stimulation at C3 vs. C4 in comparison to stimulation at C1 vs. C2 (94.8% vs. 91.0%, respectively).

A huge interindividual variability of optimal stimulation positions seems to exist in practice. Thus, a change of stimulation site may render tceMEP measurable, if stimulation at the primary site fails. If straight needle electrodes instead of corkscrew electrodes are used in small infants, good fixation is recommended, because needle electrodes have a higher risk of accidental dislocation.

Constant voltage was described in the majority of the reviewed papers. According to the guidelines of the ACNS constant voltage or constant current stimulation can be applied for tceMEP [39]. However, in 2017, Shigematsu et al. found higher success rates and higher amplitudes of tceMEP for constant voltage stimulation in comparison to constant current stimulation. Overall success rates were 86.3% for constant voltage stimulation and 68.8% for constant current stimulation [41]. These results could not be reproduced in a further study by Masuda et al. However, in contrast to constant voltage stimulation, constant current stimulation effectivity depended on the type of monophasic stimulation, thus rendering a change of stimulation polarity mandatory if tceMEP are recorded bilaterally [42]. Such practical limitations of constant current stimulation may have been observed unsystematically and may have motivated most authors to prefer constant voltage stimulation for tceMEP.

The observed high variability of the applied voltages was probably due to different skin and tissue impedances and possibly due to different sensitivity of motor cortex cells to electrical stimulation. The latter may be explained by the high variability of nervous tissue development. High variabilities of pulse widths, pulse trial counts, stimulus intervals, and applications of double or single trains suggest that these parameters can be adjusted in the ranges described, if primary stimulator settings fail to elicit sufficient tcMEPs.

### Safety considerations

Transcranial electric stimulation can cause serious complications [16, 36, 43]. Typical hazards are burns at electrode sides, bleeding at electrode sides, bite injury to the tongue, lip and ventilation tube, arrhythmia, seizures, body movement-induced injury during surgery, hypotension, respiratory/metabolic acidosis, hemorrhage, episodes, and needle-stick injuries as a hazard for operation team members [44]. These complications have to be avoided by meticulous precautions (Table 5).

**Table 5:** Precautions to avoid complications of transcranial electrical stimulation in children [16, 36, 43]

Monitoring by an experienced biomedical analyst and neurophysiologist
Structured communication with the surgeon: Communicating significant changes of tceMEP, technical problems or the impossibility to obtain tceMEP to the surgeon
Warning the surgeon prior to transcranial stimulation associated with movement
Inserting a bite block to prevent bite injuries
Using subdermal needle electrodes instead of corkscrew electrodes in patients with open skull sutures
Separating the leads for tceMEP from electrocautery cables or having them cross at right angles
Not holding the patient over needle sites when positioning
Being aware of dislodged needles especially during positioning at the beginning and at the end of surgery

Some conditions are considered relative contraindications against tceMEP by the American Clinical Neurophysiology Society (ACNS). These include epilepsy, cerebral lesions, elevated intracranial pressure, implanted devices, and convexity skull defects close to the stimulation site. In this case, the benefits of tceMEP and the potential risks of the mentioned relative contraindications have to be weighed against each other [39].

### Success rate

Predominantly in very small infants, the relatively weak synchrony of signal transduction in the immature nervous system may hamper successful eliciting of tceMEP. In young children, this immaturity of the corticospinal tract with high individual variability of corticospinal tract maturation has primarily been regarded as an obstacle to the measurement of tceMEP [9, 38]. Concordantly, Chen et al. found a significantly lower success rate for tceMEP in the lower extremities of children younger than 7 years in comparison to children and adults at an age of 7 to 64 years (55.0% vs. 70.2%, *p* < 0.01) [10].

However, stimulation protocols including spatial facilitation increased the success rate to 86% in children younger than 6 years in a study published in the same year [45]. The reviewed studies and case reports show that tceMEP can reliably be measured in very small infants thus encouraging the use of tceMEP in all age groups.

There was no difference in success rate between constant voltage pulses and constant current pulse, but only few authors used constant current stimulation, thus rendering quantitative comparison impossible (Table 4).

The improved feasibility of tceMEP in very young infants in the most recent studies may be due to increasingly refined stimulation techniques including parameter adjustment protocols rendering tceMEP feasible that could not be elicited with unadjusted primary standard stimulation parameter. A list of recommendations for modification of stimulus parameters and confounding factors is shown in Table 6.

**Table 6:** Recommendations for modification of stimulus parameters and potential confounders aiming at facilitation of tceMEP [16, 17]

Increase the pulse voltage (or current)
Increase number of stimuli
Double train stimulation instead of single train stimulation
Spatial facilitation
Obtaining tceMEPs from distal muscles with more innervation than proximal muscles may also contribute to improve tceMEP success rate
If inhalational anesthesia is used, change to total intravenous anesthesia (TIVA) can improve tceMEP measurement

### Alarm criteria

The alarm criterion of more than 50% decrease of tceMEP amplitude seems to be the most practical criterion during surgery, but changes in signal morphology and the need to increase stimulus voltage or current intensity in order to obtain reproducible tcMEP should also be considered alarm criteria. However, artifacts should be thoroughly excluded in any case of alarming changes of tcMEP. Reported criteria for significant impairment of motor pathway signal conduction were a decrease of tceMEP amplitude of more than 50%, a change of waveform from multiwave potentials to two wave potentials, or a prolongation of tceMEP latency of more than 10% [27, 30].

Two authors defined a rapid decrease of more than 80% associated with a high-risk surgical maneuver as a criteria for information of the surgical team. It was recommended that alert criteria should include more than one aspect. Besides amplitude reduction, the need for stimulus modification to obtain reproducible tceMEP and the change of tceMEP morphology from polyphasic to biphasic have been used as alert criteria [46, 47].

### Surgical context

The surgical context has to be considered in the discussion of technical aspects of tceMEP since the preferred stimulation and derivation techniques depend on the performed surgery. During brain tumor surgery, the measurement of tceMEP contributes to intraoperative neuromonitoring especially during surgery of tumors near the descending motor pathways. However, during surgery of supratentorial tumors, direct stimulation of the motor cortex is often preferred to elicit motor evoked muscle potentials. After transcranial electrical stimulation, the derivation of the D-wave is frequently feasible immediately distal of the surgical site during surgery of spinal cord tumors thus rendering direct identification of spinal cord lesions near the surgical site possible. Neuromonitoring of motor tracts during cauda equina surgery is often successfully performed by a combination of tceMEP with direct electrical stimulation of potential nervous tissue to functionally identify neural anatomical structures which are functionally ambiguous by eliciting the corresponding compound muscle action potential.

Correct interpretation of the measurement results depends on close communication between surgeon and neurophysiologist. Informing the neurophysiologist about the stage of the surgical procedure is predominantly important in critical situations with surgery near vulnerable structures. Direct view on the surgical field should be ensured for the neurophysiologist, e.g., by transmission of the surgeon’s view through the surgical microscopic on a monitor.

### Limitations

Comparability of the different study results is limited by the inhomogeneous study designs of the included publications. Most studies were retrospective and contained information from various data source types. A huge variety of equipment was used for stimulation by different authors, thus in principle also hampering comparability. There were also no generally accepted criteria for successful transcranial motor tract stimulation and tceMEP recording and even alarm criteria were not consistent. Thus, comparing different stimulation techniques and success rates quantitatively was not possible. Quantitative comparison of different stimulation techniques and success rates would have been performed, if the data obtained from the original literature would have been suitable for such an analysis. Consensus finding and standardization of these parameters would overcome these problems [26]. However, the purposed goal of our review was not a comparison of the different methodologies but to provide a quick and referenced overview over the practice of tceMEP including safety considerations without the need to screen the literature for novices and practitioners without special scientific background. tceMEP and D-wave were both included, because both methods can include transcranial electrical stimulation.

The stimulation techniques and safety considerations in this review are known from the existing literature in principle. However, this information is scattered over many publications thus rendering an overview difficult. Hence, we reviewed the technical details and safety considerations to provide a quick, but comprehensive and referenced overview over the practice of transcranial electrical stimulation in neurosurgery. Every clinician and especially novice applying tceMEP should have a detailed overview over the variety of techniques applied by experienced colleagues to know the spectrum of alternatives.

## Conclusion

The analyzed articles revealed a wide diversity of equipment, stimulation, and measurement parameters for pediatric tceMEP. Overall success rate was very high even in very young children. However, it is essential to be mindful of the recommended precautions to avoid adverse events associated with tceMEP for IONM in neurosurgery.

## Data Availability

No datasets were generated or analysed during the current study.
